# Changing patterns of nitrous oxide use and environmental awareness among Brazilian anesthesiologists: a nationwide cross-sectional survey

**DOI:** 10.1016/j.bjane.2026.844739

**Published:** 2026-02-28

**Authors:** Leopoldo Muniz da Silva, Rafael Souza Fava Nersessian, Ana Claudia Lopes Fernandes Araújo, Vanessa Melo Silva Torres, Désirée Stieven Machado, Saullo Queiroz Silveira, Fernando Nardy Bellicieri, Caroline Tukasan, Gabriel Silva dos Anjos, Helidea de Oliveira Lima, Glenio B. Mizubuti

**Affiliations:** a Rede D’Or, D’Or Institute for Research and Education (IDOR), São Paulo, SP, Brazil; b São Luiz Hospital (Rede D’Or) – CET – SBA – Hospital São Luiz ‒ CMA, São Paulo, SP, Brazil; c Vila Nova Star Hospital (Rede D’Or) ‒ CMA, São Paulo, SP, Brazil; d Queen’s University, Department of Anesthesiology and Perioperative Medicine, Kingston, Canada

**Keywords:** Anestesiologists, Climate change, General anesthesia, Nitrous oxide N_2_O, Occupational exposure

## Abstract

**Background:**

Nitrous Oxide (N_2_O) is a potent greenhouse gas with significant environmental impact and is currently the leading ozone depleting substance in use. Despite its long-standing role in anesthetic practice, data on N_2_O use and the environmental awareness of Brazilian anesthesiologists are scarce. This study aimed to describe patterns of N_2_O use and assess the level of awareness among anesthesiologists regarding its environmental impact.

**Methods:**

A nationwide cross-sectional survey was conducted among anesthesiologists in a private Brazilian hospital network. The questionnaire addressed the frequency of N_2_O use in adult and pediatric anesthesia, knowledge of its environmental effects, and attitudes toward its continued availability.

**Results:**

Of 1,238 eligible anesthesiologists, 941 completed the survey (response rate 76%). Overall, 59.3% (n = 558) reported never using N_2_O in adult patients, while 14.5% (n = 136) reported frequent or constant use. In pediatric patients, 27.5% (n = 259) reported never using N_2_O, and 38.9% (n = 366) reported frequent or constant use. Eighty percent (n = 753) indicated a reduction in N_2_O use since the beginning of their careers, and 34.5% (n = 325) were unaware of its contribution to climate change. Age and years of professional experience were directly proportional to N_2_O use in adults (p < 0.05), while awareness of its environmental impact was inversely associated with use in pediatric cases (p < 0.05).

**Conclusion:**

The survey demonstrates a shift toward reduced N_2_O use, particularly among younger anesthesiologists, although substantial use in adult practice remains. Persistent knowledge gaps highlight the importance of targeted education and policy interventions to mitigate environmental impact.

## Introduction

The global average surface temperature has increased ∼1.1°C compared to pre-industrial levels. Projections indicate that surpassing a 1.5°C increase by 2050 may significantly hinder global capacity to adapt to the impacts of climate change.^1^^,^^2^ Within healthcare systems, inhaled anesthetics, particularly Nitrous Oxide (N_2_O), are recognized as substantial contributors to greenhouse gas emissions. According to the Intergovernmental Panel on Climate Change (IPCC), N_2_O is the third most prevalent anthropogenic greenhouse gas worldwide and has a global warming potential over 100 years (GWP₁₀₀) of 273, more than double that of sevoflurane (GWP₁₀₀ = 130).^3^ This high warming potential, combined with its status as the leading ozone-depleting agent currently in use, raises concerns about its unrestricted use in healthcare. Furthermore, N_2_O stands as the leading ozone-depleting agent currently in use.^4^

Despite its environmental implications, the clinical use of N_2_O in hospital settings is not comprehensively documented and understood, and its application spans various settings, from pediatric anesthesia to labor analgesia.^5^ A longitudinal study conducted in a 1,300-bed tertiary UK hospital revealed a significant decrease in N_2_O use over 12 years, yet a quarter of the physicians surveyed did not perceive it as a meaningful contributor to climate change.^6^ In the Brazilian context, recent data from a quality improvement initiative conducted in two quaternary hospitals in São Paulo demonstrated that targeted educational and structural measures, such as disconnecting anesthesia workstations from the central N_2_O pipeline, reduced anesthesia-related N_2_O CO_2_-equivalent emissions by 82.5% without adverse clinical effects.^7^ This example shows that significant emission reduction is feasible, reinforcing the importance of understanding current clinical practice patterns and practitioners’ level of awareness as a baseline for future interventions.

Recognizing anesthesiologists’ awareness of the environmental effects of inhalational anesthetics, particularly N_2_O, is crucial for guiding sustainable strategies, integrating eco-consciousness into medical training, and promoting routine emissions reporting.^8^^,^^9^ Furthermore, mapping current patterns of N_2_O usage can support targeted interventions aimed at reducing environmental footprint through evidence-based policy development.^10^ We hypothesized that awareness among anesthesiologists regarding the environmental impact of N_2_O remains limited and that clinicians with more years of clinical practice are more likely to continue its use. This could reflect a generational shift, with newer practitioners opting for alternatives to N_2_O. This study aimed to characterize the utilization patterns of N_2_O administration among anesthesiologists employed within a private hospital network in Brazil and to evaluate their understanding of its ecological impact.

## Materials and methods

This was a nationwide, cross-sectional, observational web-based survey conducted across 72 private hospitals in Brazil between October 2 and November 2, 2024. At the time of data collection, no institutional policies or formal protocols restricting the use of N_2_O were in place in the participating hospitals. The study employed a nonprobability census sampling approach that included all anesthesiologists actively employed within the network during the data collection period who had an active institutional registration, a valid institutional email address, and departmental affiliation. Anesthesiologists who did not meet these criteria (including inactive or non-affiliated professionals) were excluded. Heads of departments were contacted directly and asked to forward the survey link, distributed via institutional email, to all eligible anesthesiologists. This process resulted in a total of 1,238 invitations. Participation was entirely voluntary and anonymous, and the survey proceeded only after informed consent was obtained.

The questionnaire was distributed through the leads of anesthesiology teams and department heads at 72 hospitals located across all five Brazilian macro-regions. These institutions represented the full hospital network operated by the private healthcare group involved in the research. While the network has national coverage, it is part of the private sector and therefore the sample does not necessarily reflect the distribution of anesthesiologists in the entire Brazilian healthcare system.

Surveys were completed via Google Forms® (Google, California, USA), enabling respondents to answer at their convenience. No identifying personal data beyond hospital affiliation was collected aside from hospital name; no individual selection or sampling techniques were applied, promoting a comprehensive and inclusive collection of perspectives. Respondents were unable to modify their previous answers, and the survey could not be paused and resumed later. Only fully completed questionnaires were considered for the final analysis. The estimated questionnaire completion time was 3 minutes. To ensure data integrity, responses originated from duplicate IP addresses were evaluated and excluded when confirmed. Additionally, responses completed in under 30 seconds from a single IP address were considered non-engaged and were removed from the dataset.

### Questionnaire design

The questionnaire was designed collaboratively by four anesthesiologists affiliated with the network’s quality improvement department. Content validity was established through review by a panel of six anesthesiologists with expertise in clinical anesthesia, environmental sustainability, and quality improvement. The survey was then conducted with ten anesthesiologists from the same hospital network who were not included in the final analysis. This pilot test evaluated the clarity of questions, estimated completion time, and technical performance of the online survey tool. Minor adjustments were made before the final distribution. The survey was tested according to previously published guidelines.^11^ To maintain objectivity and reduce interpretive bias, the questionnaire employed close-ended, multiple-choice formats.

Based on the American Society of Anesthesiologists’ (ASA) guidelines on environmentally sustainable anesthesia^12^ and other key publications addressing environmental sustainability in the perioperative setting,^1^^,^^13^^,^^14^ we developed a structured survey aimed to explore the frequency and profile of N_2_O utilization, as well as to assess the level of knowledge regarding its environmental impact. The frequency of N_2_O use in inhalational anesthesia for both adult and pediatric patients was assessed using a five-point Likert scale with the options “Never”, “Rarely”, “Sometimes”, “Often”, and “Always”. The final questionnaire comprised 17 items, divided into five sections: 1) Frequency of use in adult and pediatric patients (Questions 6‒8); 2) Fresh gas flow determination during inhalational induction (Question 9); 3) Frequency with which respondents had observed gas flow meters left open after the completion of anesthesia and when the anesthesia workstation was no longer in use (e.g., post-transport, during room turnover, or prior to initiating a subsequent anesthesia) (Question 10); 4) Perception of N_2_O environmental impact, specifically related to global warming and ozone depletion (Questions 11‒14); 5) Patterns in N_2_O clinical use and the underlying motivations for its reduction or discontinuation (Questions 15‒17). The questionnaire was developed in Portuguese (Supplementary Tables 1‒2). The primary endpoint was the self-reported frequency of N_2_O use in adult and pediatric anesthesia. Secondary endpoints included awareness of N_2_O’s environmental impact and attitudes toward its continued availability. Potential harm and/or adverse events were not applicable to this survey-based study.

This study followed the Checklist for Reporting Results of Internet E-Surveys (CHERRIES),^15^ the EQUATOR-endorsed guideline for the design and reporting of web-based surveys (see Supplementary Material for the completed checklist). All procedures involving human participants were conducted in compliance with the ethical standards established by the institutional and national research committees, as well as the principles of the Declaration of Helsinki. This study was approved by the Institutional Research Ethics Committee (Protocol n° 7,023,106; CAAE 82170024.0.0000.0087), and the approval can be verified through the public registry at Plataforma Brazil: https://plataformabrasil.saude.gov.br/visao/publico/indexPublico.jsf.

### Statistical analysis

To ensure that the number of responses would be sufficient for the planned analyses, we calculated a minimum required sample size of 294 participants using the formula n=Z²×p×(1−p)/E², where *n* represents the sample size, *Z* is the Z score corresponding to a 95% confidence level, *p* is the estimated population proportion, and *E* is the margin of error set at 5%. Anticipating an overall survey response rate between 40%‒60% (based on previous institutional survey experience and published literature on physician survey participation), we invited all eligible professionals (n = 1,238). Only fully completed questionnaires were included in the final analysis. Incomplete or partially completed questionnaires were excluded from all analyses; therefore, no imputation for missing data was performed. Responses completed in less than 30 seconds from the same IP address were considered non-engaged and excluded from the dataset. A total of six questionnaires met these criteria and were removed from the analysis.

Data normality distribution was assessed using the Shapiro-Wilk test. Categorical variables were analyzed using the Chi-Square test to compare proportions. Continuous variables were expressed as means and standard deviations for normally distributed data, or as medians with interquartile ranges otherwise. Non-parametric data were analyzed using the Kruskal-Wallis test for comparisons involving three or more categories, followed by Dunn’s post hoc method for multiple comparisons when p < 0.05, and the Mann-Whitney U test for comparisons between two categories. Adjusted associations between the self-reported frequency of N_2_O administration in adult and pediatric anesthesia (“occasionally”, “frequently” or “always”) and clinician characteristics were quantified as Odds Ratios (OR) with 95% Confidence Intervals (95% CI). Clinician characteristics included age, years of professional experience, geographic region, and the respondent’s belief that N_2_O contributes to climate change. Multivariable logistic regression was performed with simultaneous entry of all covariates. A 95% CI and p-value < 0.05 represented statistical significance. All analyses were conducted using R software version 3.4.4 (R Foundation for Statistical Computing, Austria).

## Results

**Figure. 1:**
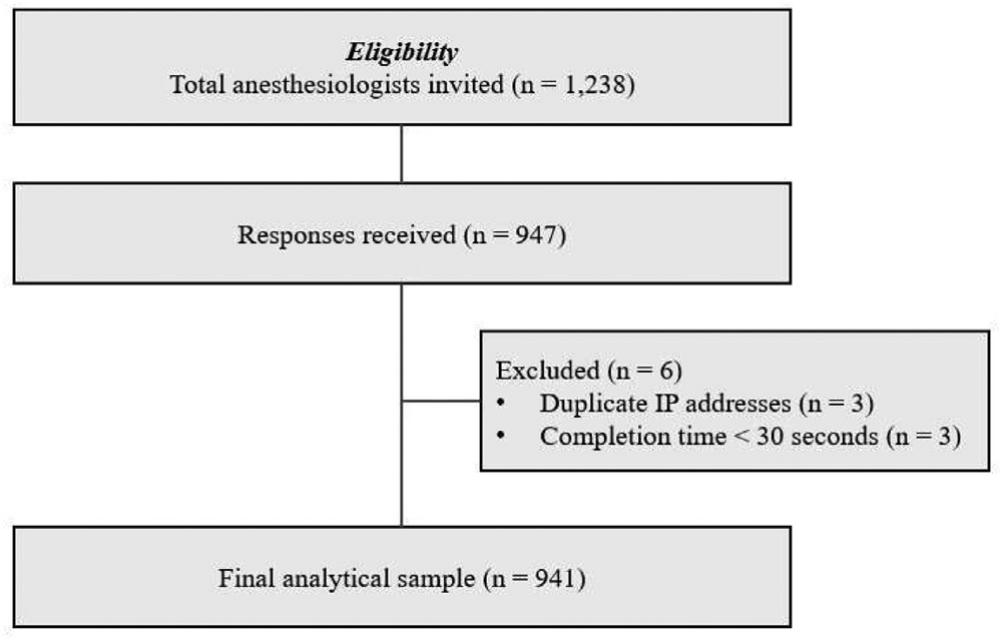
Study flow diagram showing the number of anesthesiologists invited, responses received, exclusions, and the final analytical sample.

All 72 hospitals contacted agreed to participate in the survey, and a total of 941 anesthesiologists answered the questionnaire, yielding a completion rate of 76%. The flow of participants, including invitations, exclusions, and the final analytical sample, is shown in Figure. 1. The median age of the participants was 40 (35‒49) years, with a median time of 10 (5‒21) years since completing medical residency (Supplementary Figs. 1‒2).

**Figure. 2:**
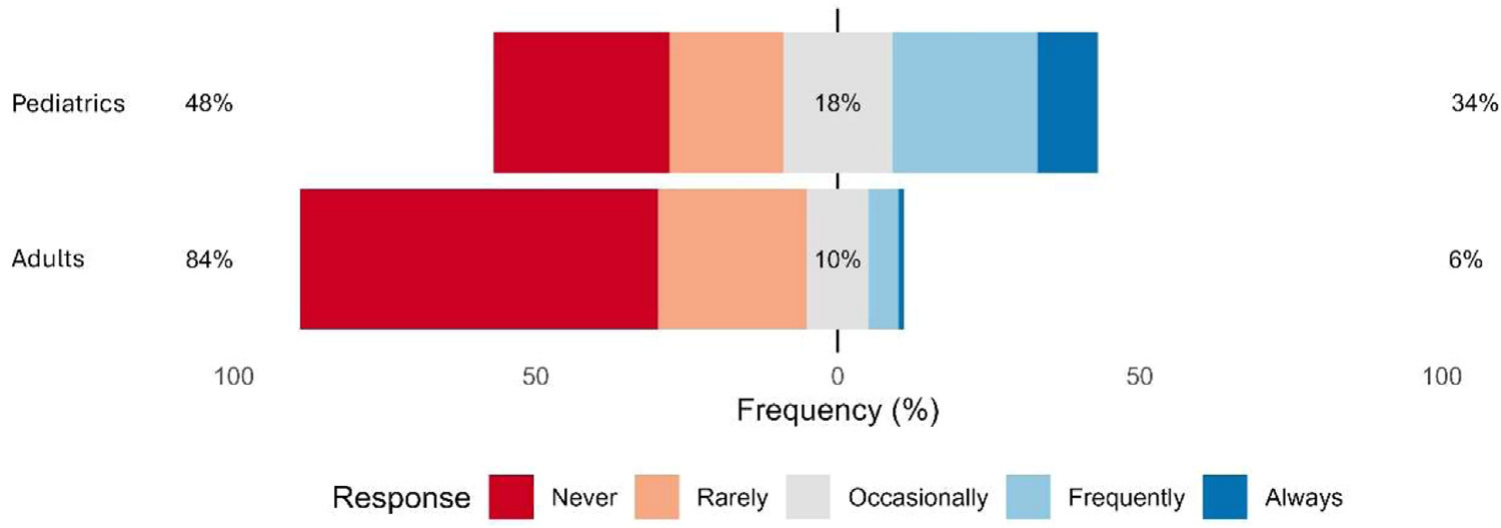
Distribution of respondents' reported frequency of N_2_O use in adults and pediatric patients.

**Figure. 3:**
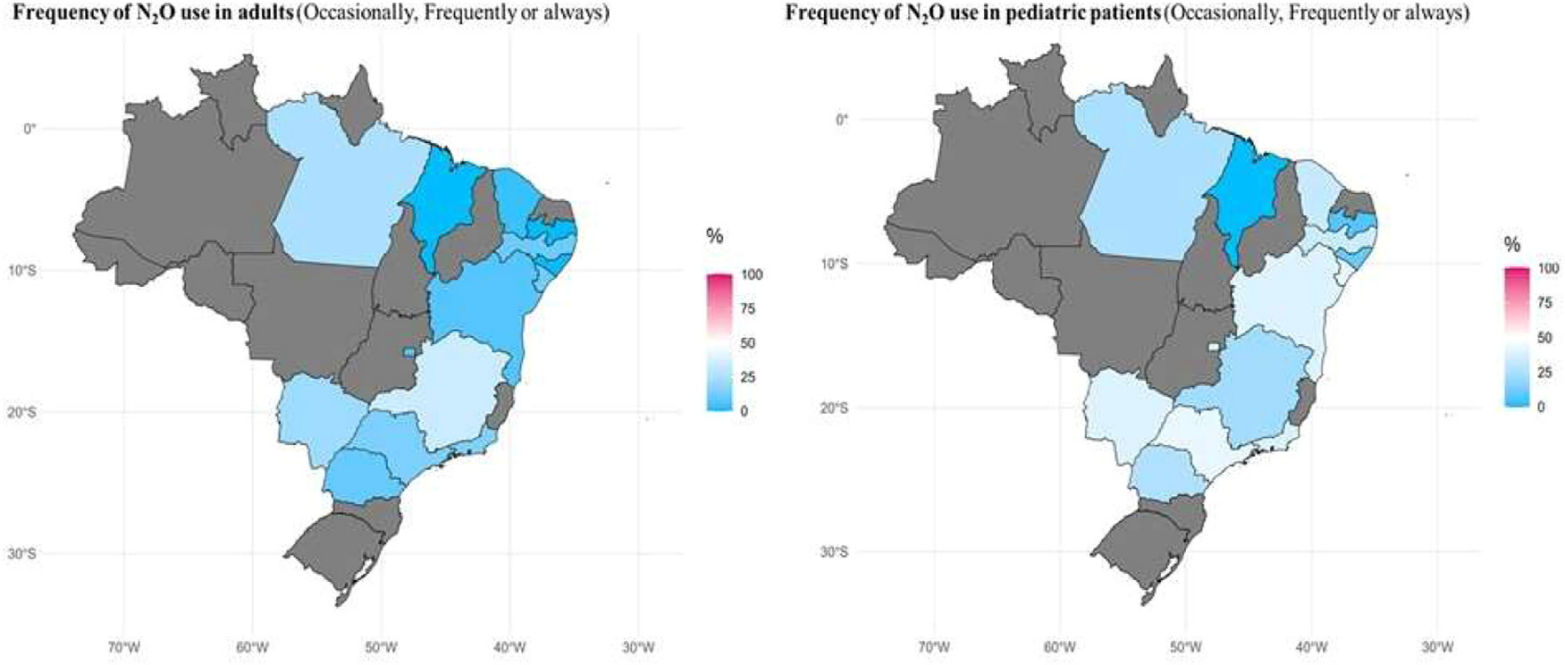
Heatmap of geographical distribution of respondents by frequency of N_2_O use in adults and pediatric patients (occasionally, frequently, or always).

A five-point Likert scale was applied to assess the frequency of N_2_O use in inhalational anesthesia for both adult and pediatric patients. Regarding adult patients, 59.3% (n = 558) of respondents reported never using N_2_O, and 5.8% (n = 55) reported frequent use (Fig. 2). Age and years of experience were directly proportional to the frequency of N_2_O use in adult patients (p < 0.05; Table 1). There was a significant difference in the proportion of responses indicating the use of N_2_O among adult patients by geographic location, with the Southeast (14.2%) and South (14.9%) regions reporting the highest rates of “always”, “frequently”, and “occasionally” use (p = 0.009) (Fig. 3; Supplementary Table 3). Among those who reported N_2_O use in adult patients (n = 383), 15.1% (n = 58) indicated its use for inhalational sedation, and 3.4% (n = 13) for labor analgesia.

**Table 1:** Frequency and profile of nitrous oxide usage, and assessment of anesthesiologists' knowledge regarding the environmental impact of this gas in a network of 72 Private Hospitals in Brazil: Differences by age (years) and years of professional experience since completion of medical residency in anesthesiology.

Variable	Overall		Age (years)		Years of experience	
	n	%	Median [IQR 25‒75]	p-value	Median [IQR 25‒75]	p-value
Frequency of N_2_O use in adults^a^				< 0.001		< 0.001
Never	558	59.3	39 [34 ‒ 45.75]		9 [4 ‒ 17]	
Rarely	231	24.6	41 [35 ‒ 49.5]		12 [5 ‒ 21.5]	
Occasionally	97	10.3	40 [36 ‒ 52]		10 [6 ‒ 23]^c^	
Frequently	46	4.9	52 [42.75 ‒ 58.75]		25 [12.25 ‒ 30]	
Always	9	1.0	55 [51 ‒ 56]^c^		30 [22 ‒ 30]^c^	
**Frequency of N_2_O use in pediatrics**^a^				0.09		0.07
Never	274	29.1	42 [36 ‒ 52]		12 [5 ‒ 24]	
Rarely	177	18.8	39 [35 ‒ 46]		9 [4 ‒ 17]	
Occasionally	170	18.1	39 [33.25 ‒ 45.75]		10 [4 ‒ 18.75]	
Frequently	226	24.0	40 [35 ‒ 47]		10 [5 ‒ 19]	
Always	94	10.0	41 [35 ‒ 52]		10 [5 ‒ 26.25]	
**FGF induction method**^a^				< 0.001		< 0.001
Patient weight (≈120 mL/kg/min)	338	35.9	42 [36 ‒ 51]		12 [5 ‒ 24.75]	
FGF of 10 L/min or more	254	27.0	38 [33.25 ‒ 45]^c^		8 [4 ‒ 16]^c^	
Clinical assessment	349	37.1	40 [35 ‒ 49]		10 [5 ‒ 21]	
**Gas flow shutdown frequency**^a^**^,^**^d^				0.02		0.08
Never	55	5.8	44 [40 ‒ 54]		14 [8 ‒ 26.5]	
Rarely	286	30.4	41 [35 ‒ 50.75]		11 [5 ‒ 21.75]	
Occasionally	327	34.7	40 [35 ‒ 47]		9 [4 ‒ 20]	
Frequently	262	27.8	40 [35 ‒ 46.75]		10 [5 ‒ 20]	
Always	11	1.2	42 [33 ‒ 52]		15 [4.5 ‒ 19]	
**N_2_O climate change Belief**^b^				0.01		0.01
No	325	34.5	41 [36 ‒ 51]		12 [5 ‒ 23]	
Yes	616	65.5	40 [34 ‒ 48]		10 [4 ‒ 20]	
**Respondents N_2_O elimination time**^a^				<0.001		< 0.001
1 year	228	24.2	45 [38 ‒ 53.25]		16 [6.75 ‒ 27]	
5 years	149	15.8	40 [35 ‒ 48]		9 [4 ‒ 18]	
15 years	134	14.2	38 [34.25 ‒ 42]^c^		8 [4 ‒ 14]^c^	
50 years	93	9.9	37 [32 ‒ 42]^c^		8 [3 ‒ 15]^c^	
Above 100 years	337	35.8	40 [35 ‒ 48]		10 [5 ‒ 21]	

a Kruskal-Wallis test.

b Mann-Whitney U test.

c Post hoc multiple comparisons using Dunn’s method FGF: Fresh Gas Flow.

d Respondents were inquired about the frequency with which they had observed gas flow meters left open after the completion of anesthesia and when the anesthesia workstation was no longer in use (for instance, following patient transport, during room preparation, or prior to initiating preparations for subsequent anesthesia).

The use of N_2_O in pediatric anesthesia was reported by 34% (n = 320) of participants (Fig. 2). Unlike in adult practice, neither age nor professional experience were associated with N_2_O use in pediatric patients (p > 0.05; Table 1). There was no difference in the frequency of N_2_O use in pediatric patients across geographic regions (p = 0.38).

Regarding the method for defining Fresh Gas Flow (FGF) during inhalational induction, FGF based on patient weight (∼120 mL/kg/min^-1^) was reported by 35.9% (n = 338), while 27% (n = 334) indicated using an FGF of 10 L/min or more, regardless of the patient's weight (Table 1). Thirty seven percent (n = 349) based their FGF on clinical assessment at the time of induction. At the conclusion of anesthesia, the practice of verifying whether the gas flow meter has been properly shut off plays a critical role in minimizing wastage, whether oxygen or inhalational agents. Respondents were inquired about how often they observed gas flow meters left open after anesthesia had ended and when the anesthesia workstation was no longer in use (e.g., post-transport, room turnover, or prior to subsequent anesthesia). Twenty-nine percent (n = 273) of anesthesiologists reported frequently or always witnessing gas flow meters left open and unused, whereas only 5.8% (n = 55) stated they had never witnessed this practice.

In response to questions about the environmental consequences of inhalational agents such as N_2_O and their contribution to climate change through global warming, 34.5% (n = 325) reported being unaware of any relationship between anesthetic technique and climate change. Seasoned anesthesiologists were more likely to be unaware of this link (p < 0.05). Only 35.8% (n = 337) correctly identified that N_2_O persists in the environment for over 100 years (Table 2).

**Table 2:** Longitudinal changes in nitrous oxide usage, access to N_2_O in operating rooms and trends in N_2_O use, in a network of 72 private hospitals in Brazil: differences by age and years of professional experience since completion of anesthesia residency.

Variable	Overall		Age (Years)		Years of experience	
	n	%	Median [IQR 25‒75]	p-value	Median [IQR 25‒75]	p-value
**Longitudinal N_2_O usage change**^a^**^,^**^c^				< 0.001		< 0.001
Maintaining	186	19.8	37 [32 ‒ 43]		6 [3 ‒ 13]	
Reduction	755	80.2	41 [36 ‒ 50]		12 [5.5 ‒ 22]	
**N_2_O free access in operating room**^a^				0.93		0.45
No	477	50.7	41 [35 ‒ 47]		10 [5 ‒ 20]	
Yes	464	49.3	40 [34.75 ‒ 50.25]		10 [4 ‒ 23]	
**Trend in the use of N_2_O**^b^				0.05		0.14
Do not use at all	249	26.5	41 [35 ‒ 50]		11 [5 ‒ 22]	
Consider eliminating	203	21.6	42 [36 ‒ 50]		11 [5 ‒ 22]	
Consider reducing	227	24.1	39 [34 ‒ 46.5]		10 [4 ‒ 18]	
Consider maintaining	262	27.8	40 [35 ‒ 49]		10 [4 ‒ 21]	
**Anesthetic technique impact from N_2_O removal**^a^				0.37		0.33
No	657	70.0	41 [35 ‒ 49]		10 [5 ‒ 21]	
Yes	284	30.0	40 [35 ‒ 48.25]		10 [4 ‒ 20]	

a Mann-Whitney U test;

b Kruskal-Wallis test.

c Comparing the current period with the period since the beginning of their training as anesthesiologists.

When comparing current practices with those at the beginning of their careers, 80% (n = 755) reported a reduction in the use of N_2_O over time. Half of the respondents, 50.7% (n = 477), stated that N_2_O should not be freely or readily available (Table 2). When asked about future use, 48% (n = 452) of respondents either reported not currently using N_2_O or indicated they intended to discontinue its use in the short term. In contrast, 27.8% (n = 262) stated they intended to continue using it. There was no significant association between these responses and either age or years of professional experience (p > 0.05).

The main reasons cited for reducing or discontinuing N_2_O use were increased incidence of nausea and vomiting (51.9%, n = 489), greater adoption of Total Intravenous Anesthesia (TIVA) (54.2%, n = 510), respiratory adverse effects such as hypoxia and atelectasis (21.1%, n = 199), its contribution to global warming and ozone layer depletion (32.2%, n = 303), pollution and occupational exposure in the operating room (37.0%, n = 349), and the perception that its clinical benefit is negligible (21.3%, n = 201). Most respondents (70%, n = 657) indicated that the withdrawal of N_2_O would not impact their anesthetic technique. There was no significant association between this perception and respondents’ age or years of professional experience regarding the impact on their anesthetic technique (p > 0.05; Table 2).

In the multivariable logistic model constructed to examine determinants of self-reported N_2_O use (“occasionally”, “frequently”, or “always”), age ≥ 50 years remained independently associated with N_2_O use in adult patients (adjusted OR = 2.99, 95% CI 1.95‒4.59) (Table 3). By contrast, the conviction that N_2_O aggravates climate change showed an inverse relation with use in adults (adjusted OR = 0.42, 95% CI 0.29‒0.61) and constituted the sole significant predictor in pediatric anesthesia (adjusted OR = 0.41, 95% CI 0.31‒0.54). Years of professional experience and geographic region did not achieve significance in either model.

**Table 3:** Adjusted odds ratios (95% CI) from multivariable logistic regression of clinician characteristics associated with self-reported nitrous oxide administration (“occasionally”, “frequently” or “always”) in adult and pediatric anesthesia.

	Self-reported nitrous oxide use (“occasionally” “frequently” or “always”)						
Characteristics	Overall n (%)	No n (%)	Yes n (%)	Crude OR (CI 95%)	p-value	Adjusted OR (CI 95%)	p-value
**Adult patients**							
**Age**							
Up to 40 years	468 (50.1)	409 (52.2)	59 (38.8)	1		1	
41 to 49	240 (25.7)	213 (27.2)	27 (17.8)	0.88 (0.53 ‒ 1.41)	0.60	0.97 (0.57 ‒ 1.60)	0.90
≥ 50 years	227 (24.3)	161 (20.6)	66 (43.4)	2.84 (1.91 ‒ 4.23)	< 0.001	2.99 (1.95 ‒ 4.59)	< 0.001
**Years of experience**							
Less than 10 years	58 (6.2)	49 (6.3)	9 (5.9)	1		1	
10 years or more	877 (93.8)	734 (93.7)	143 (94.1)	1.06 (0.53 ‒ 2.35)	0.87	0.75 (0.36 ‒ 1.74)	0.47
**Geographic Region**							
North	4 (0.3)	3 (0.3)	1 (0.7)	1		1	
Northeast	334 (25.7)	309 (26.9)	25 (16.5)	0.24 (0.03 ‒ 5.00)	0.22	0.19 (0.02 ‒ 4.38)	0.19
Central-West	169 (13)	156 (13.6)	13 (8.6)	0.25 (0.03 ‒ 5.25)	0.24	0.31 (0.03 ‒ 7.29)	0.37
Southeast	767 (59)	658 (57.3)	109 (71.7)	0.50 (0.06 ‒ 10.10)	0.54	0.50 (0.04 ‒ 11.09)	0.57
South	27 (2.1)	23 (2)	4 (2.6)	0.52 (0.05 ‒ 12.00)	0.60	0.56 (0.04 ‒ 14.44)	0.67
**N_2_O Climate Change Belief**							
No	324 (34.7)	244 (31.2)	80 (52.6)	1		1	
Yes	611 (65.4)	539 (68.8)	72 (47.4)	0.41 (0.29 ‒ 0.58)	< 0.001	0.42 (0.29 ‒ 0.61)	< 0.001
**Pediatric patients**							
**Age**							
Up to 40 years	468 (50.1)	210 (46.9)	258 (53)	1		1	
41 to 49	240 (25.7)	124 (27.7)	116 (23.8)	0.76 (0.56 ‒ 1.04)	0.08	0.78 (0.56 ‒ 1.08)	0.13
≥ 50 years	227 (24.3)	114 (25.5)	113 (23.2)	0.81 (0.59 ‒ 1.11)	0.18	0.76 (0.54 ‒ 1.07)	0.11
**Years of experience**							
Less than 10 years	58 (6.2)	25 (5.6)	33 (6.8)	1		1	
10 years or more	877 (93.8)	423 (94.4)	454 (93.2)	0.81 (0.47 ‒ 1.39)	0.44	0.93 (0.52 ‒ 1.63)	0.80
**Geographic Region**							
North	4 (0.3)	3 (0.4)	1 (0.2)	1		1	
Northeast	334 (25.7)	218 (26.8)	116 (23.8)	1.60 (0.20 ‒ 32.47)	0.68	1.66 (0.15 ‒ 37.61)	0.68
Central-West	169 (13)	113 (13.9)	56 (11.5)	1.49 (0.19 ‒ 30.43)	0.73	2.05 (0.18 ‒ 46.89)	0.57
Southeast	767 (59)	464 (57)	303 (62.2)	1.96 (0.25 ‒ 39.70)	0.56	2.85 (0.25 ‒ 64.23)	0.40
South	27 (2.1)	16 (2)	11 (2.3)	2.06 (0.23 ‒ 44.77)	0.55	2.78 (0.21 ‒ 69.59)	0.44
**N_2_O Climate Change Belief**							
No	324 (34.7)	110 (24.6)	214 (43.9)	1		1	
Yes	611 (65.4)	338 (75.5)	273 (56.1)	0.42 (0.31 ‒ 0.55)	< 0.001	0.41 (0.31 ‒ 0.54)	< 0.001

CI, Confidence intervals; OR, odds ratio.

## Discussion

This study characterized patterns of N_2_O use in anesthesia and identified gaps in anesthesiologists’ awareness of its environmental impact. Eighty percent of respondents reported a reduction in use since the beginning of their careers, and nearly half expressed reservations about its unrestricted availability. In contrast, 34.5% of anesthesiologists were unaware of its role in climate change. These patterns may be related to differences in training exposure, evolving clinical preferences, or increasing awareness of sustainability issues in more recent generations of practitioners. Given the cross-sectional nature of this study, these factors should be interpreted as possible contributors rather than causal determinants.

Understanding these usage patterns is essential for informing strategies aimed at sustainable environmental impact. Our results support the development of targeted practice protocols aimed at curtailing N_2_O use, particularly in cases where its clinical benefits are outweighed by alternative anesthetic agents/techniques. Additionally, reports of frequent gas flow meter misuse after procedures (29%) suggest inefficiencies that warrant attention, as they may result in unnecessary emissions and resource waste.^16^ If N_2_O consumption is minimal and not critical for clinical practice, healthcare leaders should reassess the need to maintain N_2_O supply infrastructure and focus on interventions aimed at optimizing consumption patterns.^17^^,^^18^

In a Brazilian quality improvement initiative for adult patients, baseline data over the 16-week pre-intervention period indicated an average N_2_O usage rate of 11%, decreasing to 2% post-intervention.^7^ Considering these results, the use of N_2_O in adult patients remains a prevalent practice, and a cultural shift is necessary to reduce its usage in more restricted contexts, such as the inhalational induction of pediatric patients.^6^^,^^8^ In pediatric anesthesia, targeted quality improvement interventions have shown substantial environmental benefits. In a 20-month project involving over 33,000 inhalational inductions, reducing N_2_O use from 80% to less than 20% and lowering FGF by 28% was achieved through education, expert engagement, and visual reminders, without affecting induction times or patient behavior.^8^ This suggests that even minor regulatory or logistical barriers could substantially reduce N_2_O use.^5^^,^^19^

The practice of using high FGF during inhalational induction was also analyzed. High FGF is often employed to hasten induction or to compensate for mask leaks during facemask ventilation. However, this practice results in the consumption of large quantities of volatile agents, almost all of which are wasted directly into the environment.^20^ Strikingly, less than 40% of anesthesiologists regulate FGF based on the patient's weight, even though exceeding the minute-ventilation does not meaningfully accelerate induction and leads to significant volatile agent consumption.^21^ Since minute-ventilation is correlated with the patient's weight, the Society for Pediatric Anesthesia (SPA) recommends using a weight-based minimum safe and effective FGF of 0.15 L/min/kg during inhalational induction.^22^ This recommendation includes a safety margin to ensure no rebreathing and accounts for differences between anesthesia delivery systems and individual patient variation.^8^ However, our data demonstrated that pediatric anesthesiologists often used fixed high FGF (e.g., 10  L/min of oxygen and N_2_O, with or without sevoflurane), regardless of patient's size, contradicting best practices and exacerbating environmental impact. Coupled with prior studies indicating that elevated FGF during induction is correlated with increased sevoflurane consumption and higher FGF levels throughout cases, these results underscore the importance of adopting an optimized and environmentally sustainable approach to anesthetic management.^14^^,^^23^

Data from Italy align with our observations, showing a gradual reduction in the use of inhalational agents and a persistent lack of awareness of environmental issues among anesthesiologists.^24^ Indeed, the majority of anesthesiologists stated that TIVA could replace anesthetic gases in more than 40% of cases, while only 5% believed this could happen in less than 20% of cases.^24^ Similarly, 65% of anesthesiologists were partially or completely unaware of the environmental impact of anesthetic gases, and the use of high FGF remains a common practice in inhalational anesthesia.^24^ Similar to the reduction observed in our study, a longitudinal evaluation in a 1,300-bed tertiary hospital in the United Kingdom demonstrated a marked decline in N_2_O use over 12 years, indicating that the decrease in N_2_O utilization is not limited to Brazil; however, in that UK cohort, one quarter of physicians did not recognize it as a significant contributor to climate change.^6^

Senior anesthesiologists were more likely to report frequent N_2_O use in adult patients, although no significant generational difference was observed in pediatric use. There were no differences in age or professional experience regarding the perception of its impact on anesthetic technique or the tendency to reduce its use. This suggests that, despite the historical use of N_2_O among more senior anesthesiologists, they may be open to reducing its utilization, with unwanted clinical effects being the main driver for limiting its use. This contrasts with results from the UK, where environmental impact was the primary reason for reducing N_2_O use.^6^ Such differences imply that “green anesthesia” remains in early stages in Brazil, despite global acknowledgement of climate change as “the biggest threat to global health in the 21^st^ century”.

Our results delineate a marked generational gradient in practice patterns: anesthesiologists aged ≥ 50-years were nearly three times more likely to administer N_2_O to adult patients, whereas acknowledging its climate impact was inversely associated with use and remained the sole significant determinant in pediatric anesthesia. One plausible explanation is that sustainable practice is still insufficiently embedded in medical training: the environmental knowledge gap among senior educators hampers the transmission of sustainability principles to trainees, creating a persistent barrier to behavior change. Accordingly, environmental stewardship should be formally integrated into residency curricula and continuing professional development for practicing anesthesiologists, as multiple studies across different countries have already advocated.^24^^,^^25^

Several factors may influence the discontinuation of N_2_O use in Brazil, operating as either barriers or drivers of change. Cultural barriers include the long-standing tradition of using N_2_O for pediatric inhalational induction and the absence of environmental sustainability as a structured component of anesthesiology training. Institutional barriers involve the persistence of central pipeline supply systems (which are often faulty/leaky) and the lack of infrastructure modifications needed to reduce systemic losses. Educational barriers stem from limited exposure to concepts of sustainable anesthesia and occupational safety related to anesthetic gases during both residency and continuing professional development. On the other hand, important drivers for reducing N_2_O use include the growing global and national emphasis on climate change mitigation, the increasing availability and familiarity with TIVA, and awareness of the occupational hazards associated with waste anesthetic gases. Evidence from successful Brazilian initiatives, such as the 82.5% reduction in N_2_O emissions achieved in two quaternary hospitals through targeted education and pipeline disconnection, reinforces the feasibility of large-scale change.^7^ These national experiences, in line with the global trend toward N_2_O phase-out, highlight that meaningful reductions are possible when clinical engagement is combined with infrastructure and policy interventions.^7^^,^^9^^,^^26^^,^^27^

Despite consistent evidence that occupational exposure to inhalational anesthetics, particularly in operating rooms without active scavenging systems, exceeds international safety limits and poses health risks,^28^^,^^29^ only 37% of respondents in our study cited pollution or occupational exposure as a reason to reduce N_2_O use. This underestimation is particularly concerning since longitudinal evidence shows that such exposures are not merely theoretical risks.^30^ A prospective study evaluating young physicians before, during, and at the end of their medical residency demonstrated that working in unavenged operating rooms leads to substantial exposure to inhalational anesthetics, resulting in lipid and protein oxidation, oxidative DNA damage, impairment of antioxidant systems, modulation of gene expression involved in DNA repair, and measurable genotoxic effects.^31^ Our results suggest a significant gap in awareness or a tendency among anesthesiologists to underestimate the occupational hazards associated with waste anesthetic gases in the operating room and/or global environment.

The results of this study have relevant implications for policy, education, and clinical practice in Brazil. In the educational sphere, environmental stewardship and occupational safety related to anesthetic gases should be incorporated into undergraduate, residency, and continuing medical education curricula to build awareness from the earliest stages of training. In clinical practice, dissemination of best practices for optimizing FGF, greater adoption of TIVA when clinically appropriate, and institution-led quality improvement programs can collectively contribute to reducing N_2_O emissions. Aligning these strategies with international sustainability goals offers a pathway for Brazilian anesthesiology to achieve measurable environmental impact reduction without compromising patient care.^12^^,^^24^^,^^25^

Contemporary guidance from professional societies and health systems increasingly supports the reduction of nitrous oxide use and the decommissioning of centralized piped N_2_O systems, primarily due to their disproportionate contribution to emissions through chronic leakage. The World Federation of Societies of Anaesthesiologists and partner organizations have endorsed principles of environmentally sustainable anesthesia that include minimizing or eliminating routine N_2_O use, optimizing fresh gas flows, and addressing avoidable infrastructural losses.^1^ More recent consensus from the United Kingdom and Ireland recommend the removal of piped N_2_O manifolds and transition to point-of-use cylinders, driven by data indicating that a substantial proportion of N_2_O supplied via pipelines may be lost to leakage before reaching patients, thereby generating emissions with minimal clinical benefit.^6^^,^^18^ Similar action plans and toolkits from NHS systems and other jurisdictions highlight N_2_O mitigation as a priority, emphasizing leak detection, decommissioning of underused manifolds, and alignment with net-zero strategies.^17^^,^^32^ These positions are consistent with analyses showing that N_2_O is a long-lived greenhouse gas and a leading ozone-depleting substance, and that infrastructural waste rather than conscious clinical use can account for a large share of its footprint.^4^ In this context, the reduction in self-reported N_2_O use observed among Brazilian anesthesiologists ‒ particularly the limited perceived dependence on N_2_O for routine practice ‒ supports the feasibility of targeted institutional policies and infrastructure reviews aimed at rationalizing or discontinuing N_2_O supply where clinically appropriate, while maintaining patient safety.

Our results should also be interpreted in light of the evolving Brazilian regulatory framework for medical gases. ANVISA RDC n° 870/2024 and Normative Instruction n° 301/2024, n° 301/2024 classify medicinal gases, including N_2_O, as regulated medicinal products and establish contemporary requirements for notification, registration, and quality assurance, with defined implementation timelines.^33^^,^^34^ Within this framework, hospitals are expected to ensure that centralized medical gas systems and gas mixers comply with technical standards such as ABNT NBR 12188 (centralized medical gas pipeline systems) and ABNT NBR 15882 (oxygen-nitrous oxide gas mixers). Decisions to maintain, rationalize, or decommission centralized N_2_O infrastructure should follow these standards, be guided by documented low clinical demand, robust safety assessments, and leak prevention, and be integrated into broader environmental sustainability and patient safety strategies.^35^^,^^36^ While our survey did not directly audit regulatory compliance or engineering practices, the observed trend toward reduced N_2_O use among Brazilian anesthesiologists is consistent with and supportive of this regulatory direction.

This study has limitations. First, the web-based design and voluntary participation introduce potential selection bias, as the sample was drawn from a convenience census of eligible anesthesiologists and relied on self-reporting, which may not reflect actual clinical behavior. Second, although the sample size calculation was appropriately performed and largely exceeded, with 941 completed questionnaires and a high response rate of 76%, the study employed a nonprobability sampling approach restricted to anesthesiologists from a private hospital network in Brazil. This may limit the generalizability of the results to other healthcare systems, including public hospitals and rural settings. Third, the questionnaire was not formally validated through psychometric testing, which may affect the reliability and reproducibility of the results, although content review by experts was performed before distribution. Fourth, social desirability bias may have influenced responses, particularly for items related to environmentally responsible behaviors. Fifth, the absence of objective data or verification regarding the actual use of N_2_O limits the ability to validate self-reported information. Sixth, the study did not collect information on the surgical case mix of each participating hospital, which could influence N_2_O usage patterns, as hospitals with surgical profiles less likely to require N_2_O, such as those without pediatric, obstetric, or specific inhalational sedation cases, might have contributed disproportionately to lower reported usage rates. Lastly, the cross-sectional design and absence of longitudinal follow-up prevent the assessment of temporal trends and causality, and other potentially relevant variables, such as institutional policies, availability of TIVA, or local anesthetic protocols, were not examined.

## Conclusion

This national survey identified reduced N_2_O use among younger anesthesiologists, suggesting evolving practice patterns potentially driven by changes in training, increased familiarity with alternative anesthetic techniques, and heightened awareness of occupational and environmental considerations. However, a substantial proportion of practitioners, particularly older anesthesiologists, remain unaware of N_2_O’s long atmospheric lifetime and its role in global warming. Addressing this knowledge gap through targeted educational strategies, coupled with evidence based institutional and national policies, could support the optimization of N_2_O use while reducing its environmental footprint. The fact that half of the respondents questioned its continued unrestricted availability underscores the opportunity for policy makers to act. Future research using validated instruments and nationally representative samples will be essential to confirm these results and inform sustainable anesthesia practices in Brazil and beyond.

## Conflicts of interest

The authors declare no conflicts of interest and confirm that no artificial intelligence tools were used in the conception, analysis, or preparation of this manuscript.

## Appendix Supplementary materials

Image, application 1

## Footnotes

Supplementary material associated with this article can be found in the online version at doi:10.1016/j.bjane.2026.844739.
