# Supplementary material for: Changing patterns of nitrous oxide use and environmental awareness among Brazilian anesthesiologists: a nationwide cross-sectional survey
Source: Braz J Anesthesiol. 2026 Feb 28;76(3):844739. doi: 10.1016/j.bjane.2026.844739 (PMC13085057; doi:10.1016/j.bjane.2026.844739)

## BJAN-D-25-00373

***Changing patterns of nitrous oxide use and environmental awareness among Brazilian anesthesiologists: a nationwide cross-sectional survey***

**Supplementary Material**

Silva et al.

| Table of contents | |  |
| --- | --- | --- |
| Supplementary Files | Figure &Table Headings | Page |
| Supplementary Table 1 | Questionnaire (Portuguese version). | 3 |
| Supplementary Table 2 | Questionnaire (English version) | 6 |
| Supplementary Figure 1 | Age (years) of respondents - Histogram | 9 |
| Supplementary Figure 2 | Time since completing medical residency (years) of respondents - Histogram | 9 |
| Supplementary Table 3 | Geographical Distribution of Respondents by Frequency of N_2_O Use in Adults and Pediatric Patients (Occasionally, Frequently, or Always). | 10 |
| **Supplementary Table 4** | Checklist for Reporting Results of Internet E-Surveys (CHERRIES) | 11 |
|  | Ethics Approval Statement | 15 |

## Supplementary Table 1. Questionnaire (Portuguese version)

# Questionário sobre Padrões de Utilização do Óxido Nitroso

1) Unidade hospitalar participante: __________

2) Estado: __________

3) Categoria profissional:
( ) Médico anestesiologista

4) Idade: __________

5) Quanto tempo de formação (anos) em anestesiologia (após o término da residência até a data atual)? __________

**Frequência de uso em pacientes adultos e pediátricos**

6) Com que frequência você costuma utilizar o óxido nitroso em anestesia inalatória para pacientes adultos?
( ) Nunca
( ) Raramente
( ) Às vezes
( ) Muitas vezes
( ) Sempre

7) Com que frequência você costuma utilizar o óxido nitroso em anestesia inalatória para pacientes pediátricos?
( ) Nunca
( ) Raramente
( ) Às vezes
( ) Muitas vezes
( ) Sempre

8) Assinale os tipos de técnica nas quais você utiliza óxido nitroso na sua prática clínica (permite mais de uma resposta):
( ) Anestesia inalatória em neonatos
( ) Anestesia inalatória em pediatria
( ) Anestesia inalatória em adultos
( ) Analgesia de parto
( ) Sedação com anestésicos inalatórios
( ) Não utilizo nunca

**Método para definir o fluxo de gases frescos na indução inalatória em anestesia pediátrica**
9) Como você definiria o fluxo de gases frescos na indução inalatória de pacientes pediátricos com utilização do óxido nitroso?
( ) Baseado em um fluxo de gases frascos de cerca de 10 L por minuto ou mais
( ) Baseado em estratificação por faixas de peso (120 mL.kg^-1^.min^-1^)
( ) Baseado na avaliação clínica no momento da indução

**Frequência de observação de fluxômetros de gases deixados abertos após a conclusão da anestesia e quando o aparelho de anestesia já não estava em uso**

10) Você já presenciou o término de alguma anestesia no qual o fluxômetro de gases do aparelho de anestesia ficou aberto por engano (Após o transporte do paciente após o término da anestesia, durante a limpeza e preparação da sala para próxima cirurgia ou durante os preparativos para a anestesia subsequente)?
( ) Nunca
( ) Raramente
( ) Às vezes
( ) Muitas vezes
( ) Sempre

**Sustentabilidade**

11) Você considera que o uso do óxido nitroso em anestesia contribui para mudanças climáticas relacionadas ao aquecimento global?
( ) Sim
( ) Não

12) Ao utilizar óxido nitroso durante sua anestesia inalatória, em quanto tempo você considera que este gás utilizado será degradado e eliminado do meio ambiente?
( ) 1 ano
( ) 5 anos
( ) 15 anos
( ) 50 anos
( ) Acima de 100 anos

13) Você considera que o óxido nitroso deva estar disponível de maneira livre e permanente nas salas cirúrgicas para o uso clínico?
( ) Sim
( ) Não

14) Você sentiria impacto na sua técnica anestésica se o uso do óxido nitroso fosse abolido do centro cirúrgico?
( ) Sim
( ) Não

**Tendências de utilização do óxido nitroso**

15) Em relação à utilização do óxido nitroso na sua prática clínica atual:
( ) Comparado ao início da minha formação, reduzi o uso
( ) Comparado ao início da minha formação, mantive ou aumentei o uso

16) Quais os motivos que melhor justificam a redução do uso, caso esta tenha sido a sua resposta para pergunta anterior (permite mais de uma resposta):
( ) Emissões de gases de efeito estufa/ destruição da camada de ozônio
( ) Aumento de náuseas e vômitos
( ) Aumento do uso de anestesia venosa total
( ) Poluição no centro cirúrgico/exposição ocupacional
( ) Efeitos adversos respiratórios (hipóxia, atelectasia)
( ) Efeito não relevante do ponto de vista clínico

17) Assinale sua conduta futura em relação ao uso do óxido nitroso:
( ) Considero manter
( ) Considero reduzir
( ) Considero eliminar
( ) Não utilizo de forma alguma

## Supplementary Table 2. Questionnaire (English version)

1) Participating hospital unit: __________

2) State: __________

3) Professional category:
( ) Anesthesiologist

4) Age: __________

5) Years of professional experience in anesthesiology (from residency completion to the present date): __________

**Frequency of Use in Adult and Pediatric Patients**

6) How frequently do you use nitrous oxide for inhalational anesthesia in adult patients?
( ) Never
( ) Rarely
( ) Sometimes
( ) Often
( ) Always

7) How frequently do you use nitrous oxide for inhalational anesthesia in pediatric patients?
( ) Never
( ) Rarely
( ) Sometimes
( ) Often
( ) Always

8) Indicate the techniques in which you use nitrous oxide in your clinical practice (multiple answers allowed):
( ) Inhalational anesthesia in neonates
( ) Inhalational anesthesia in pediatrics
( ) Inhalational anesthesia in adults
( ) Labor analgesia
( ) Sedation with inhalational anesthetics
( ) I never use it
 **Method for Determining Fresh Gas Flow in Pediatric Inhalational Induction**
9) How do you determine fresh gas flow during inhalational induction in pediatric patients using nitrous oxide?
( ) Based on a fresh gas flow (approximately 10 L per minute or more)
( ) Based on weight-stratified flow adjustment (120 mL.kg^-1^.min^-1^)
( ) Based on clinical assessment at the time of induction

**Frequency of Observing Flowmeters Left Open After Anesthesia Completion**

10) Have you ever witnessed an anesthesia case where the flowmeter remained unintentionally open after the procedure?
( ) Never
( ) Rarely
( ) Sometimes
( ) Often
( ) Always

**Sustainability**

11) Do you believe that the use of nitrous oxide in anesthesia contributes to climate change?
( ) Yes
( ) No

12) How long do you estimate it will take for nitrous oxide to degrade and be eliminated from the environment?
( ) 1 year
( ) 5 years
( ) 15 years
( ) 50 years
( ) More than 100 years

13) Should nitrous oxide be freely and permanently available in operating rooms?
( ) Yes
( ) No

14) Would your anesthetic technique be affected if nitrous oxide were withdrawn?
( ) Yes
( ) No

**Trends in Nitrous Oxide Utilization**

15) Compared to the beginning of my training, I have:
( ) Reduced its use
( ) Maintained or increased its use

16) Reasons for reducing use (if applicable):
( ) Greenhouse gas emissions
( ) Increased nausea and vomiting
( ) Increased use of TIVA
( ) OR pollution/occupational exposure
( ) Respiratory adverse effects
( ) Clinically irrelevant effect

17) Future approach to nitrous oxide use:
( ) Maintain
( ) Reduce
( ) Eliminate
( ) Do not use it at all

**
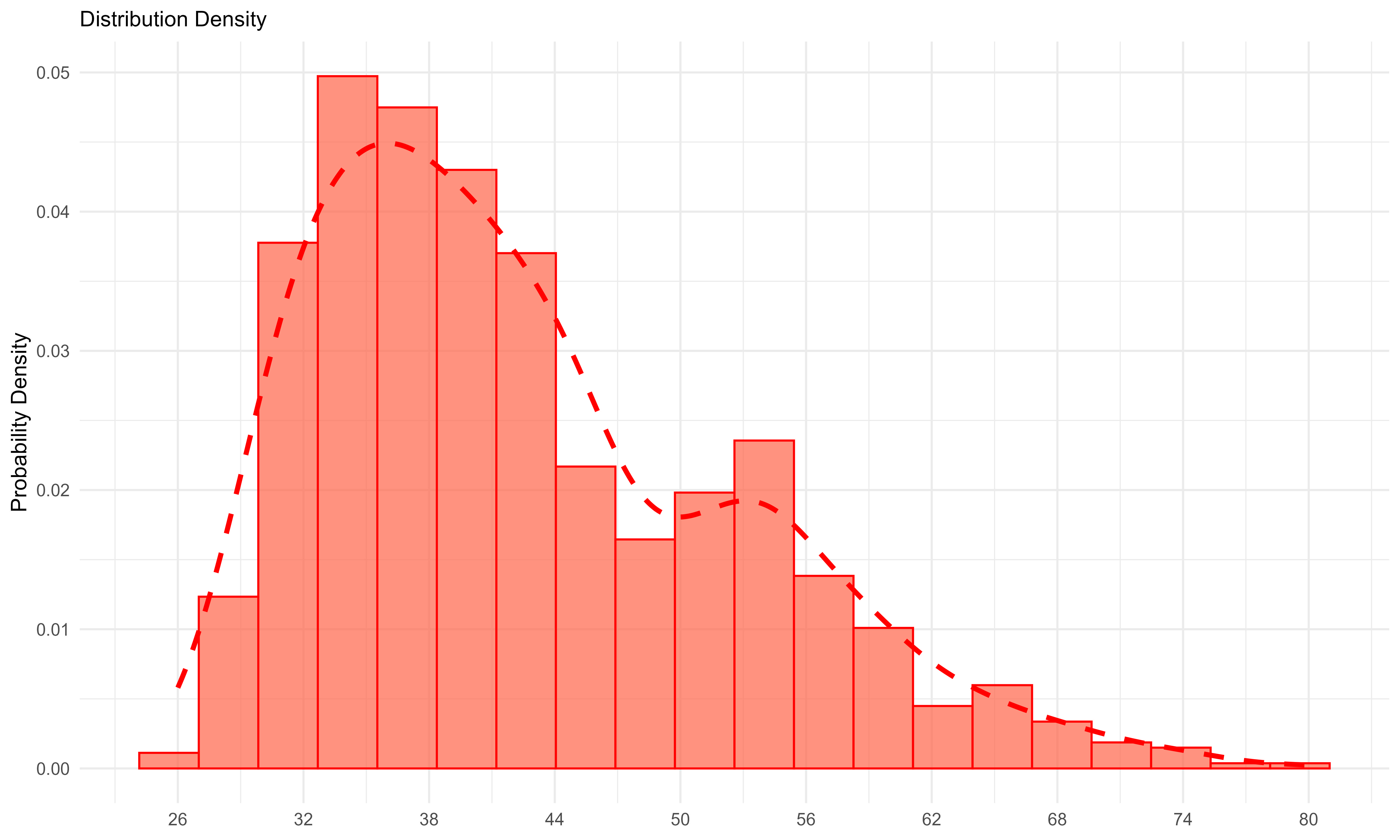
**

**Supplementary Figure 1.** Age (years) of respondents - Histogram.


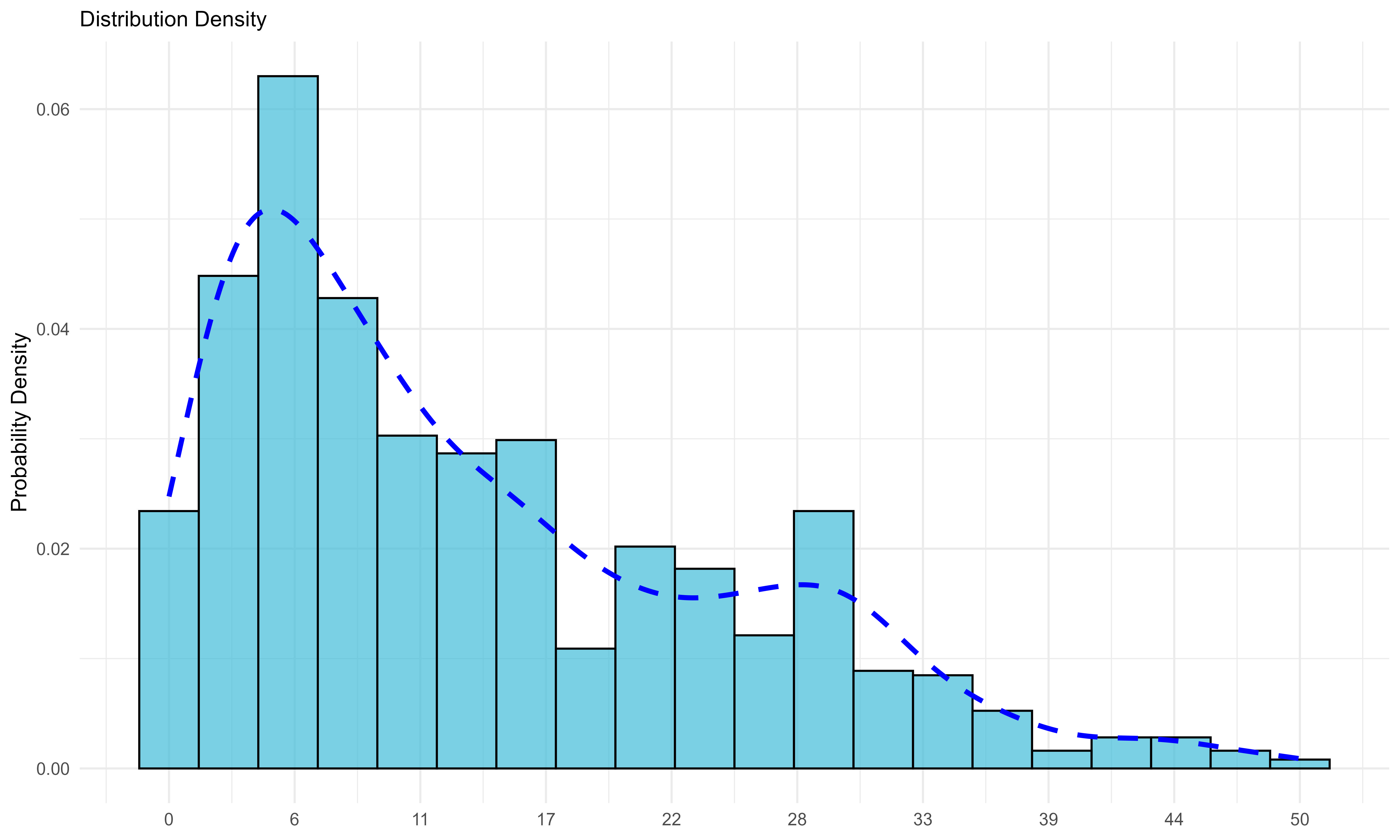


**Supplementary Figure 2.** Time since completing medical residency (years) of respondents - Histogram.

## Supplementary Table 3. Geographical Distribution of Respondents by Frequency of N_2_O Use in Adults and Pediatric Patients (Occasionally, Frequently, or Always).

| **Geographical region** | **Overall** | **Frequency of N_2_O use in adults n (%)*** | **p-value** | **Frequency of N_2_O use in pediatrics n (%)*** | **p-value** |
| --- | --- | --- | --- | --- | --- |
|  |  |  | 0.007 |  | 0.38 |
| North | 4 (100.0) | 1 (25.0) |  | 1 (25.0) |  |
| Northeast | 334 (100.0) | 25 (7.5) |  | 116 (34.7) |  |
| Midwest | 169 (100.0) | 13 (7.7) |  | 56 (33.1) |  |
| Southeast | 767 (100.0) | 109 (14.2) |  | 303 (39.5) |  |
| South | 27 (100.0) | 4 (14.8) |  | 11 (40.7) |  |

## * Frequency of N_2_O Use in Adults and Pediatric Patients (Occasionally, Frequently, or Always).

**Supplementary Table 4.** Checklist for Reporting Results of Internet E-Surveys (CHERRIES).

| ***Checklist Item*** | ***Explanation*** | ***Page Number*** |
| --- | --- | --- |
| Describe survey design | Describe target population, sample frame. Is the sample a convenience sample? (In “open” surveys this is most likely.) | 10-13 |
| IRB approval | Mention whether the study has been approved by an IRB. | 13 |
| Informed consent | Describe the informed consent process. Where were the participants told the length of time of the survey, which data were stored and where and for how long, who the investigator was, and the purpose of the study? | 13 |
| Data protection | If any personal information was collected or stored, describe what mechanisms were used to protect unauthorized access. | 11 |
| Development and testing | State how the survey was developed, including whether the usability and technical functionality of the electronic questionnaire had been tested before fielding the questionnaire. | 10-13 |
| Open survey versus closed survey | An “open survey” is a survey open for each visitor of a site, while a closed survey is only open to a sample which the investigator knows (password-protected survey). | 10-13 |
| Contact mode | Indicate whether or not the initial contact with the potential participants was made on the Internet. (Investigators may also send out questionnaires by mail and allow for Web-based data entry.) | 10-13 |
| Advertising the survey | How/where was the survey announced or advertised? Some examples are offline media (newspapers), or online (mailing lists – If yes, which ones?) or banner ads (Where were these banner ads posted and what did they look like?). It is important to know the wording of the announcement as it will heavily influence who chooses to participate. Ideally the survey announcement should be published as an appendix. | 10-13 |
| Web/E-mail | State the type of e-survey (eg, one posted on a Website, or one sent out through e-mail). If it is an e-mail survey, were the responses entered manually into a database, or was there an automatic method for capturing responses? | 11-12 |
| Context | Describe the Website (for mailing list/newsgroup) in which the survey was posted. What is the Website about, who is visiting it, what are visitors normally looking for? Discuss to what degree the content of the Website could pre-select the sample or influence the results. For example, a survey about vaccination on a anti-immunization Website will have different results from a Web survey conducted on a government Website | 11-12 |
| Mandatory/voluntary | Was it a mandatory survey to be filled in by every visitor who wanted to enter the Website, or was it a voluntary survey? | 11-12 |
| Incentives | Were any incentives offered (eg, monetary, prizes, or non-monetary incentives such as an offer to provide the survey results)? | NA |
| Time/Date | In what timeframe were the data collected? | 10-12 |
| Randomization of items or questionnaires | To prevent biases items can be randomized or alternated. | NA |
| Adaptive questioning | Use adaptive questioning (certain items, or only conditionally displayed based on responses to other items) to reduce number and complexity of the questions. | NA |
| Number of Items | What was the number of questionnaire items per page? The number of items is an important factor for the completion rate. | 10-12 |
| Number of screens (pages) | Over how many pages was the questionnaire distributed? The number of items is an important factor for the completion rate. | 10-12 |
| Completeness check | It is technically possible to do consistency or completeness checks before the questionnaire is submitted. Was this done, and if “yes”, how (usually JAVAScript)? An alternative is to check for completeness after the questionnaire has been submitted (and highlight mandatory items). If this has been done, it should be reported. All items should provide a non-response option such as “not applicable” or “rather not say”, and selection of one response option should be enforced. | 11 |
| Review step | State whether respondents were able to review and change their answers (eg, through a Back button or a Review step which displays a summary of the responses and asks the respondents if they are correct). | 11 |
| Unique site visitor | If you provide view rates or participation rates, you need to define how you determined a unique visitor. There are different techniques available, based on IP addresses or cookies or both. | NA |
| View rate (Ratio of unique survey visitors/unique site visitors) | Requires counting unique visitors to the first page of the survey, divided by the number of unique site visitors (not page views!). It is not unusual to have view rates of less than 0.1 % if the survey is voluntary. | NA |
| Participation rate (Ratio of unique visitors who agreed to participate/unique first survey page visitors) | Count the unique number of people who filled in the first survey page (or agreed to participate, for example by checking a checkbox), divided by visitors who visit the first page of the survey (or the informed consents page, if present). This can also be called “recruitment” rate. | NA |
| Completion rate (Ratio of users who finished the survey/users who agreed to participate) | The number of people submitting the last questionnaire page, divided by the number of people who agreed to participate (or submitted the first survey page). This is only relevant if there is a separate “informed consent” page or if the survey goes over several pages. This is a measure for attrition. Note that “completion” can involve leaving questionnaire items blank. This is not a measure for how completely questionnaires were filled in. (If you need a measure for this, use the word “completeness rate”.) | NA |
| Cookies used | Indicate whether cookies were used to assign a unique user identifier to each client computer. If so, mention the page on which the cookie was set and read, and how long the cookie was valid. Were duplicate entries avoided by preventing users access to the survey twice; or were duplicate database entries having the same user ID eliminated before analysis? In the latter case, which entries were kept for analysis (eg, the first entry or the most recent)? | NA |
| IP check | Indicate whether the IP address of the client computer was used to identify potential duplicate entries from the same user. If so, mention the period of time for which no two entries from the same IP address were allowed (eg, 24 hours). Were duplicate entries avoided by preventing users with the same IP address access to the survey twice; or were duplicate database entries having the same IP address within a given period of time eliminated before analysis? If the latter, which entries were kept for analysis (eg, the first entry or the most recent)? | NA |
| Log file analysis | Indicate whether other techniques to analyze the log file for identification of multiple entries were used. If so, please describe. | NA |
| Registration | In “closed” (non-open) surveys, users need to login first and it is easier to prevent duplicate entries from the same user. Describe how this was done. For example, was the survey never displayed a second time once the user had filled it in, or was the username stored together with the survey results and later eliminated? If the latter, which entries were kept for analysis (eg, the first entry or the most recent)? | NA |
| Handling of incomplete questionnaires | Were only completed questionnaires analyzed? Were questionnaires which terminated early (where, for example, users did not go through all questionnaire pages) also analyzed? | 11 |
| Questionnaires submitted with an atypical timestamp | Some investigators may measure the time people needed to fill in a questionnaire and exclude questionnaires that were submitted too soon. Specify the timeframe that was used as a cut-off point, and describe how this point was determined. | NA |
| Statistical correction | Indicate whether any methods such as weighting of items or propensity scores have been used to adjust for the non-representative sample; if so, please describe the methods. | NA |

This checklist has been modified from Eysenbach G. Improving the quality of Web surveys: the Checklist for Reporting Results of Internet E-Surveys (CHERRIES). J Med Internet Res. 2004 Sep 29;6(3):e34 [erratum in J Med Internet Res. 2012; 14(1): e8.]. Article available at [https://www.jmir.org/2004/3/e34](https://www.jmir.org/2004/3/e34/)/; erratum available at <https://www.jmir.org/2012/1/e8/>.

**Ethics Approval Statement**

This investigation was approved by the São Luiz & Rede D’Or Hospitals Research Ethics Committee (Protocol number 7,023,106; CAAE 82170024.0.0000.0087). The approval can be verified through the official public registry at Plataforma Brasil:

<https://plataformabrasil.saude.gov.br/visao/publico/indexPublico.jsf>.


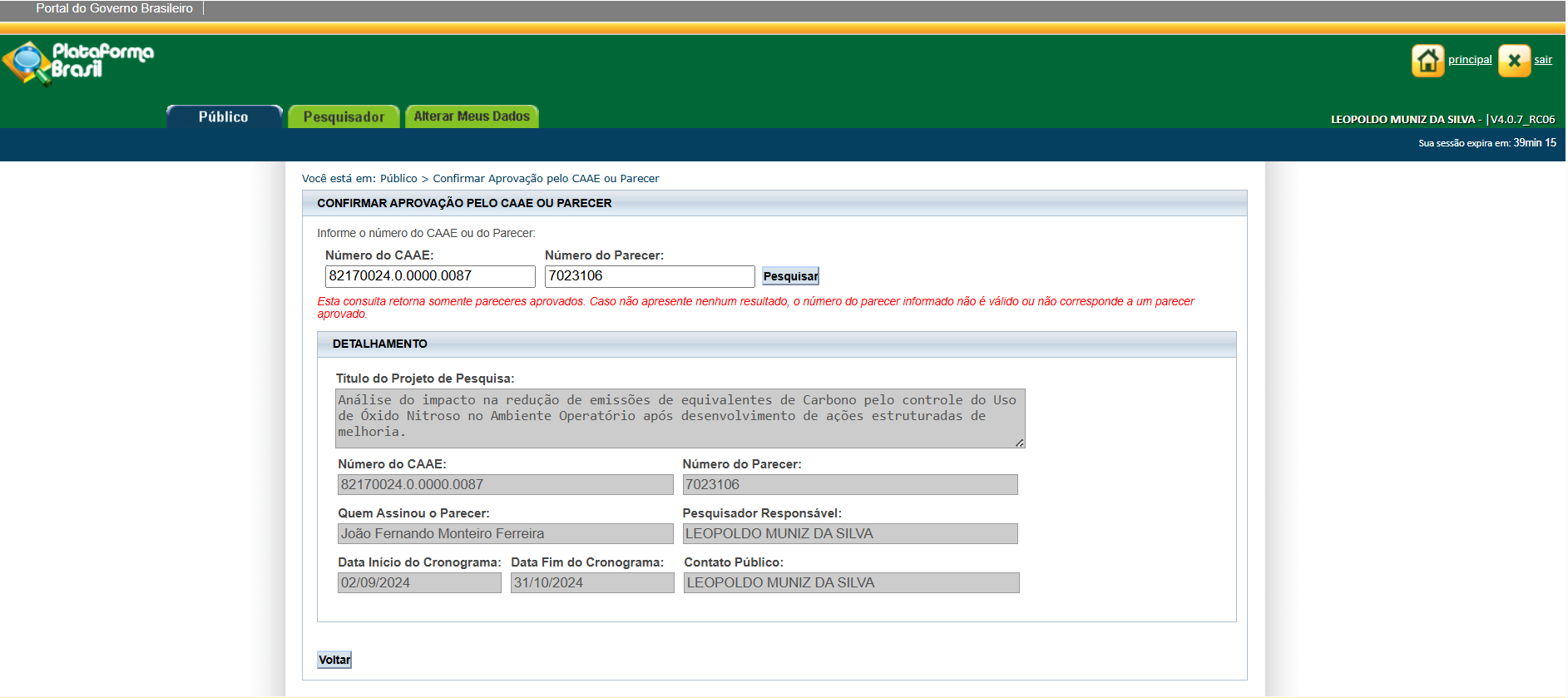

Supplement: Supplementary file 1 [file mmc1.docx]
